# Navigation-guided endoscopic lumbar decompression on foramen and lateral recess in advanced scoliosis

**DOI:** 10.3171/2024.1.FOCVID23195

**Published:** 2024-04-01

**Authors:** Dimas Rahman Setiawan, Enrico Giordan, Changik Lee, Chan Woong Park, Phattareeya Pholprajug, Jin-Sung Kim

**Affiliations:** 1 Spine Center, Department of Neurosurgery, Seoul St. Mary’s Hospital, College of Medicine, The Catholic University of Korea, Seoul, South Korea;; 2 Neurosurgery Department, Medistra Hospital, Jakarta, Indonesia;; 3 Neurosurgical Department, AULSS2 Marca Trevigiana, Treviso, Italy; and; 4 Orthopedics Department, Rayong Hospital, Rayong, Thailand

**Keywords:** MISS, lumbar endoscopic surgery, navigation guided, decompression, scoliosis

## Abstract

An 84-year-old woman presented with left leg radiating pain for 18 months and a numeric rating scale score of 8. From examination, motoric on left knee extension was grade 4, with dysesthesia and numbness along the left anterolateral thigh. Imaging showed left L3–4 foraminal and lateral recess stenosis with severe-degree scoliosis. The patient underwent navigation-guided endoscopic transforaminal foraminotomy and lateral recess decompression on the left L3–4 level with a good outcome. Three-years’ follow-up showed a well-maintained clinical outcome and coronal sagittal balance. This video explores navigation-guided endoscopic lumbar decompression for neural compression in advanced scoliosis. Further research is encouraged to establish long-term efficacy.

The video can be found here: https://stream.cadmore.media/r10.3171/2024.1.FOCVID23195

## Transcript

The authors present a case of navigation-guided endoscopic lumbar decompression on foramen and lateral recess in advanced scoliosis.

### 0:28 Case Description.

An 84-year-old female patient presented with left leg radiating pain (anterolateral thigh) for 18 months. On examination, motoric on left knee extension was grade 4, with dysesthesia and numbness along the left anterolateral thigh. This video shows how the patient walks, and she is complaining of left thigh pain. BMD T-score was −3.4.

### 0:56 Radiological Imaging.

X-ray spine showed a nonprogressive scoliosis from 2011 to 2019. Last Cobb’s angle was 58.7°. Preoperative whole-body spine x-ray shows neutral coronal balance with positive sagittal balance (34.45 mm). CT and MRI shows left severe foraminal and lateral recess stenosis on L3–4 level with diffuse bulging disc and ligamentum flavum thickening. Left lateral recess might pinch L4 traversing root. CT and MRI on L4–5 and L5–S1 level also show foraminal stenosis but not as severe as L3–4 level.

### 1:41 Diagnosis and Treatment Options.

Diagnosis was made based on clinical and radiological findings. Patient’s symptoms on L3–4 dermatome and from imaging show severe foraminal and lateral recess stenosis on left L3–4 level in severe-degree scoliosis. Diagnostic root block is performed to confirm pain generators. The main purpose for treatment is treating the pain generator.^1^ Endoscopic decompression on the left L3–4 level is preferred over deformity correction. We preferred decompression surgery rather than fusion surgery because of several factors in the patient, such as old age, poor bone quality, medical comorbidities,^2^ and pain generator seems due to compression of left L3–4 nerve root.

### 2:24 Patient Positioning and Navigation Preparation.

Patient underwent endoscopic transforaminal foraminotomy and lateral recess decompression on the left L3–4 level, under general anesthesia and in a prone position. We use a navigation system to enhance accuracy of planned decompression targets. Reference frame was attached to patient’s back using tape. This frame serves as a reference point for the navigation system throughout the procedure.^3^ Left L3–4 foramen was checked with O-arm navigation system (before making an incision).

### 2:54 Skin Incision and Endoscope Preparation.

A vertical 1-cm incision was made at desired level, 4–5 cm from the midline. After incision was made, obturator was inserted. We use a 5.5-mm working channel. Before introducing the endoscope, we reconfirm left L3–4 foramen with O-arm navigation system. We calibrate and attach navigation tracker to our surgical instruments. This ensures that the system accurately tracks the position and orientation of these instruments in real time during the surgery.^3^ After that, we introduce the endoscope and connect water system. We use a 20° endoscope. Navigation tracker is attached to endoscope instrument.

### 3:38 Tissue Exposure.

This is the initial endoscopic view of paraspinal muscle after docking. Muscle dissection was performed using a radiofrequency probe to resect the overlying muscle to identify the bone landmarks. By using a beveled edge of the working channel, we can dissect using a rotating maneuver.^4^

### 4:01 Drilling.

After muscle dissection, the bony structures of pars and SAP (superior articular processes) were exposed. Lateral aspect of pars was drilled. Further dissection targeted the junction of the lateral border of the pars and the transverse process (TP). The inferior edge of the L3 transverse process was meticulously dissected. Subsequently, drilling of the transverse and pars junction was conducted using a diamond burr, including the inferior part of the L3 transverse process. After drilling, wider space was obtained for introducing endoscope into the foramen. To make foraminal space wider, we drilled lateral border of pars, medial part of transverse process and pars junction L3. Resection of transverse process and soft tissue was performed using Kerrison punches. In the video, we can see the proper technique for securely holding and rotating the endoscope with left hand. Additionally, right index and middle fingers were positioned on the endoscope to control the depth of the drill.

Then, pars was drilled to expose the dorsal surface of nerve. Posterior aspect of nerve root was exposed.

### 5:20 Root Expose and Decompression.

Using dissector, foraminal ligament was detached from the transverse process and we can assess the edge of pars drilled. Resection of the transverse process was performed using Kerrison punches to expose the nerve root.

Since there was a severe adhesion, care was taken when removing the transverse process and pars over the nerve root. We assess the extent of transverse process resection to help ensure adequate removal to relieve compression on the nerve roots and avoid overresection. The caudal part of SAP was drilled to decompress lateral recess, and superficial layer of ligamentum flavum was cut. Ligamentum flavum was removed in piecemeal fashion. Ligamentum flavum over the traversing root is also being resected. Dissection and resection of ligamentum flavum was performed at the exit portion of traversing root and exiting root. A bit more of caudal and medial part of transverse process is removed to unroof the exiting root from proximal to distal part. Then, to decompress traversing root, we dissect and remove the ligamentum flavum also in piecemeal fashion. Fibrous band and neural structure in the shoulder region of traversing root are being dissected. Also, decompressing the distal portion of exiting root was performed. The remnant ligamentum flavum in the distal portion of the exiting root was removed using a Kerrison and shrunk using a radiofrequency probe. Using curved hook, lateral and medial wall of pedicle was palpated to confirm sufficient decompression of the proximal portion of the exiting nerve root.

We perform annuloplasty using radiofrequency probe for annular sealing.^5^ Remnant ligamentum flavum was removed for decompressing distal portion of the nerve root. Ligamentum flavum was dissected and excised to decompress traversing root.

### 8:07 Lateral Recess Decompression.

Finally, lateral recess decompression was accomplished, ensuring a comprehensive approach to traversing root decompression. Exiting root and traversing root was decompressed well.

### 8:18 Skin Closure.

Subcutaneous suture was done on skin incision.

### 8:22 Surgical Summary.

Length of surgery was 1 hour and 30 minutes. Estimated blood loss was 20 ml.

### 8:29 Postoperative Outcomes.

The patient exhibited improved left leg pain postoperatively, with the ability to ambulate just a few hours after surgery. Notably, there were no intraoperative complications. (The patient was discharged on postoperative day 3.)

### 8:40 Postoperative Imaging.

Postoperative x-ray and CT scans revealed an increased foraminal space, affirming the positive impact of the surgical intervention.^6^

### 8:50 Follow-Up Status.

The 3-year postoperative follow-up indicates that the pain has disappeared, and the patient has achieved an excellent outcome and satisfaction score. Whole-spine x-ray also shows well-maintained coronal and sagittal alignment over 3 years after surgery.

## Disclosures

Dr. Kim reported being a consultant for Richard Wolf, GmbH, and Elliquence, LLC.

## Author Contributions

Primary surgeon: Kim. Assistant surgeon: Lee, Park. Editing and drafting the video and abstract: Setiawan. Critically revising the work: Setiawan, Lee. Reviewed submitted version of the work: Kim, Setiawan, Giordan, Lee, Pholprajug. Approved the final version of the work on behalf of all authors: Kim. Supervision: Kim.

## Supplemental Information

### Patient Informed Consent

The necessary patient informed consent was obtained in this study.
